# *PLD3* is accumulated on neuritic plaques in Alzheimer’s disease brains

**DOI:** 10.1186/s13195-014-0070-5

**Published:** 2014-11-02

**Authors:** Jun-ichi Satoh, Yoshihiro Kino, Yoji Yamamoto, Natsuki Kawana, Tsuyoshi Ishida, Yuko Saito, Kunimasa Arima

**Affiliations:** 1 Department of Bioinformatics and Molecular Neuropathology, Meiji, Pharmaceutical University, 2-522-1 Noshio, Kiyose 204-8588, Tokyo, Japan; 2 Department of Pathology and Laboratory Medicine, Kohnodai Hospital, NCGM, 1-7-1 Kohnodai, Ichikawa 272-8516, Chiba, Japan; 3 Department of Laboratory Medicine, National Center Hospital, NCNP, 4-1-1 Ogawahigashi, Kodaira 187-8502, Tokyo, Japan; 4 Department of Psychiatry, Komoro Kogen Hospital, Kou 4598, Komoro 384-8540, Nagano, Japan

## Abstract

**Introduction:**

Recently, a whole-exome sequencing (WES) study showed that a rare variant rs145999145 composed of p.Val232Met located in exon 7 of the phospholipase D3 (*PLD3*) gene confers a doubled risk for late-onset Alzheimer’s disease (AD). Knockdown of PLD3 elevates the levels of extracellular amyloid-beta (Aβ), suggesting that PLD3 acts as a negative regulator of Aβ precursor protein (APP) processing. However, the precise cellular location and distribution of PLD3 in AD brains remain largely unknown.

**Methods:**

By quantitative RT-PCR (qPCR), western blot, immunohistochemistry, and bioinformatics analysis, we studied PLD3 expression patterns and levels in a series of AD and control brains, including amyotrophic lateral sclerosis, Parkinson’s disease, multiple system atrophy, and non-neurological cases.

**Results:**

The levels of PLD3 mRNA and protein expression were reduced modestly in AD brains, compared with those in non-AD brains. In all brains, PLD3 was expressed constitutively in cortical neurons, hippocampal pyramidal and granular neurons but not in glial cells. Notably, PLD3 immunoreactivity was accumulated on neuritic plaques in AD brains. We identified the human granulin (*GRN*) gene encoding progranulin (PRGN) as one of most significant genes coexpressed with PLD3 by bioinformatics database search. PLD3 was actually coexpressed and interacted with PGRN both in cultured cells *in vitro* and in AD brains *in vivo*.

**Conclusions:**

We identified an intense accumulation of PLD3 on neuritic plaques coexpressed with PGRN in AD brains, suggesting that PLD3 plays a key role in the pathological processes of AD.

## Introduction

Alzheimer’s disease (AD) is characterized by the hallmark pathology, comprised of widespread amyloid-beta (Aβ) deposition, neurofibrillary tangle formation, and extensive neurodegeneration in the brain. The complex interaction between multiple genetic and environmental factors causes AD pathology [1]. Previously, genome-wide association studies identified more than 20 common variants with lower penetrance and smaller risk for late-onset AD [2]. Recently, whole-exome sequencing studies discovered rare functional variants located in Aβ precursor protein (*APP*), triggering receptor expressed on myeloid cells 2 (*TREM2*), and phospholipase D3 (*PLD3*) genes that exhibit a much greater contribution to protection or development of AD [3]-[5].

Phospholipase D (PLD), a phospholipid-modifying enzyme whose activation is triggered by growth factors, hormones, and neurotransmitters, catalyzes the hydrolysis of phosphatidylcholine to generate free choline and phosphatidic acid, the latter of which is converted into diacylglycerol by phosphatidic acid phosphatases [6],[7]. Both phosphatidic acid and diacylglycerol, by acting as a second messenger, play a key role in membrane trafficking, cytoskeleton reorganization, receptor-mediated endocytosis, exocytosis, cell growth, differentiation, migration, and regulation of the cell cycle. PLD is directly activated by phosphatidylinositol 4,5-bisphosphate, ADP-ribosylation factor, Rho family small GTPases, and protein kinase C [6],[7].

Accumulating evidence showed a significant crosstalk between PLD, APP, and presenilin-1 with relevance to amyloidogenesis in AD brains [8]. The biochemical activity of PLD is decreased in AD brains [9]. The Aβ25–35 fragment stimulates PLD activity in LA-N-2 human neuroblastoma cells [10]. Aβ42 peptide internalized via formayl-peptide receptor-like-1 (FPRL1) activates PLD in cultured rat astrocytes and microglia [11]. The human genome contained six distinct genes encoding PLD enzymes, designated as *PLD1* to *PLD6*. Among them, both PLD1 and PLD2 regulate neurite outgrowth [12]. PLD1 is translocated to the Golgi/trans-Golgi complex by interacting with the cytoplasmic loop region of presenilin-1, and thereby negatively regulates Aβ production [13]. Furthermore, PLD1 promotes trafficking and cell surface accumulation of presenilin-1 in an APP-independent manner [14]. Overexpression of APP elevates PLD2 activity in P19 mouse embryonic carcinoma cells [15]. PLD2 is required for the synaptotoxic action of Aβ oligomers [16]. Thus, nearly all studies have previously focused on a role of PLD1 and PLD2 in AD pathology.

PLD3 is a poorly characterized member of the PLD superfamily, expressed abundantly in the central nervous system, predominantly located in endoplasmic reticulum (ER) in a subpopulation of neurons in the mouse brain [17],[18]. PLD3 protein consists of a transmembrane domain and two PLD phosphodiesterase domains with two copies of the catalytically essential HXKXXXXD/E (HKD) motif, the hallmark characteristic of the PLD family, although neither canonical activity nor substrate for PLD3 has so far been identified [17],[18]. A recent study showed that a single nucleotide variant numbered rs145999145 in exon 7 of the human *PLD3* gene on chromosome 19q13.2, comprised of p.Val232Met located in the phosphodiesterase domain close to the HKD motif, confers a doubled risk for development of late-onset AD in a large case–control series [5]. PLD3 interacts with APP in cultured cells, and overexpression of PLD3 reduces intracellular APP and extracellular Aβ42 and Aβ40, whereas knockdown of PLD3 elevates the levels of extracellular Aβ42 and Aβ40, suggesting that PLD3 acts as a negative regulator of APP processing [5]. However, the precise cellular location and distribution of PLD3 in AD brains remain largely unknown.

To study the role of PLD3 in AD pathology, we characterized PLD3 expression patterns and levels in a series of AD and non-AD brains by quantitative reverse transcription-polymerase chain reaction (qPCR), western blot, immunohistochemistry, and bioinformatics analysis. We found that the PLD3 mRNA and protein levels are reduced modestly in AD brains. Regardless of reduced levels of PLD3, it is accumulated on neuritic plaques coexpressed with the neurodegeneration-related molecule progranulin (PGRN).

## Materials and methods

### Human brain tissues

For immunohistochemical studies, serial sections of the frontal cortex and the hippocampus were prepared from autopsied brains of 10 sporadic AD patients, composed of five men and five women with a mean age of 70 ± 8 years, and 13 non-AD patients, composed of six men and seven women with a mean age of 74 ± 8 years, as described previously [19]. The non-AD group includes four normal subjects who died of non-neurological causes, three patients with sporadic Parkinson’s disease, four patients with sporadic amyotrophic lateral sclerosis, and two patients with sporadic multiple system atrophy. The demographic profile of the cases is summarized in Table 1. All AD cases satisfied the Consortium to Establish a Registry for Alzheimer’s Disease criteria for diagnosis of definite AD [20]. They were categorized into stage C of amyloid deposition and stage VI of neurofibrillary degeneration, following the Braak staging system [21]. Additional cases for which frozen brain samples were available were included for biochemical and genetic studies (Table 1). Autopsies were performed at the National Center Hospital, National Center of Neurology and Psychiatry, Japan or Kohnodai Hospital, National Center for Global Health and Medicine, Japan. The comprehensive examination of autopsied brains by three established neuropathologists (KA, YS, TI) validated the pathological diagnosis. In all cases, written informed consent was obtained. The Ethics Committee of the National Center of Neurology and Psychiatry for the Human Brain Research, the Ethics Committee of the National Center for Global Health and Medicine on the Research Use of Human Samples, and the Human Research Ethics Committee of the Meiji Pharmaceutical University approved the present study.

**Table 1:** Demographic profile of the cases examined in the present study

**Case number**	**IHC**	**qPCR/WB**	**Cause of death**	**Brain weight (g)**	**Postmortem interval (hours)**	**Braak staging (amyloid deposition/neurofibrillary degeneration)**	**p.V232M variant**	**c.1326G > A variant**
NC1	+	+	Acute myocardial infarction	1,130	1.4	A/II	V/V	G/G
NC2	+	+	Acute myocardial infarction	1,350	1.6	0/II	V/V	G/G
NC3	+	+	Lung cancer	1,060	3.9	A/II	V/V	G/G
NC4	+	+	Dissecting aortic aneurysm	1,400	4.8	A/I	V/V	G/G
AD1	+	+	Pneumonia	1,000	1.1	C/VI	V/V	G/G
AD2	+	+	Pneumonia	1,230	14	C/VI	V/V	G/G
AD3	+	+	Pneumonia	1,220	10.5	C/VI	V/V	G/G
AD4	+	+	Pneumonia	1,240	8.1	C/VI	V/V	G/G
AD5	ND	+	Lung cancer	1,090	4.5	C/VI	V/V	G/G
AD6	+	+	Pulmonary infarction	840	3	C/VI	V/V	G/G
AD7	ND	+	Respiratory failure by aspiration	1,200	3.8	B/IV	V/V	G/G
AD8	+	ND	Pneumonia	1,060	8	C/VI	ND	ND
AD9	+	ND	Pneumonia	970	NA	C/VI	ND	ND
AD10	+	ND	Pneumonia	870	1	C/VI	ND	ND
AD11	+	ND	Lung cancer	1,125	14.5	C/VI	ND	ND
AD12	+	ND	Multiple organ failure	775	13	C/VI	ND	ND
PD1	ND	+	Pneumonia	1,330	9.5	B/IV	V/V	G/G
PD2	+	+	Pneumonia	1,130	2.5	B/II	V/V	G/G
PD3	+	+	Respiratory failure by aspiration	910	2.5	B/II	V/V	G/G
PD4	ND	+	Colon cancer	1,430	4	A/I	V/V	G/G
PD5	+	ND	Pneumonia	1,320	9.3	C/III	ND	ND
ALS1	+	+	Respiratory failure	1,480	10.5	0/0	V/V	G/G
ALS2	+	+	Respiratory failure	1,090	1.3	0/I	V/V	G/G
ALS3	+	+	Respiratory failure	1,560	3	0/I	V/V	G/G
ALS4	+	+	Respiratory failure	1,320	10	0/II	V/V	G/G
ALS5	ND	+	Respiratory failure	1,360	2.5	B/I	V/V	G/G
ALS6	ND	+	Respiratory failure	1,600	13	B/I	V/V	G/G
MSA1	+	ND	Pneumonia, septicemia	1,040	1.5	0/I	ND	ND
MSA2	+	ND	Pneumonia	1,090	12	A/I	ND	ND

Demographic profile of the cases processed for immunohistochemistry (IHC), quantitative reverse transcription-polymerase chain reaction (qPCR), and western blot (WB) is shown with the case number, the cause of death, brain weight, the postmortem interval, the Braak Staging (amyloid deposition/neurofibrillary degeneration), p.Val232Met variant of rs145999145, and c.1326G > A variant of rs4819. AD, Alzheimer’s disease; ALS, amyotrophic lateral sclerosis; MSA, multiple system atrophy; NA, information not available; NC, non-neurological causes; ND, not determined; PD, Parkinson’s disease.

### Immunohistochemistry

The brain tissues were fixed with 4% paraformaldehyde and embedded in paraffin. Tissue sections were deparaffinized and heat treated in 10 mM citrate sodium buffer, pH 6.0 by autoclaving them at 110°C for 15 minutes in a temperature-controlled pressure chamber (Biocare Medical, Concord, CA, USA). For Aβ immunolabeling, tissue sections were exposed to formic acid at room temperature for 5 minutes. They were incubated at room temperature for 15 minutes with 3% hydrogen peroxide-containing methanol to block the activity of endogenous peroxidase. They were incubated with phosphate-buffered saline containing 10% normal goat serum at room temperature for 15 minutes to block nonspecific staining. The tissue sections were then incubated at 4°C overnight with a rabbit polyclonal anti-PLD3 antibody raised against a peptide spanning amino acid residues 93 to 218 of the human PLD3 protein at a concentration of 0.067 μg/ml (HPA012800; Sigma, St Louis, MO, USA). The specificity of HPA012800 was validated by western blot analysis of recombinant Flag-tagged PLD3 protein expressed in HeLa cells (Additional file 1). After washing with phosphate-buffered saline, the tissue sections were labeled at room temperature for 30 minutes with a peroxidase-conjugated secondary antibody (Nichirei, Tokyo, Japan), followed by incubation with diaminobenzidine tetrahydrochloride substrate (Vector, Burlingame, CA, USA). Finally, they were processed for a counterstain with hematoxylin. To prepare negative controls, the tissue sections were incubated with the primary antibody preabsorbed by recombinant PLD3 fragment spanning amino acid residues 93 to 218 (homemade).

For double-labeling, the tissue sections were initially stained with a mouse monoclonal anti-Aβ11-28 antibody (1 μg/ml, 12B2; Immunobiological Laboratory, Gunma, Japan), a mouse monoclonal anti-PHF-tau antibody (0.25 μg/ml, AT8; Thermo Scientific, Rockford, IL, USA), a mouse monoclonal anti-progranulin antibody (3.3 μg/ml, PG359-7; Adipogen, Liestal, Switzerland), a mouse monoclonal anti-GFAP antibody (prediluted, GA5; Nichirei), or a mouse monoclonal anti-CD68 antibody (prediluted, KP1; Dako, Tokyo, Japan). They were then incubated with an alkaline phosphatase-conjugated secondary antibody (Nichirei), and colorized with a Warp Red chromogen (Biocare Medical). After autoclaving the sections, they were relabeled with the anti-PLD3 antibody HPA012800, followed by incubation with a peroxidase-conjugated secondary antibody (Nichirei), and colorized with diaminobenzidine tetrahydrochloride substrate (Vector).

### Reverse transcriptase-polymerase chain reaction analysis

Total cellular RNA was extracted from human neural cell lines and tissues using TRIZOL (Invitrogen, Carlsbad, CA, USA). RNA treated with DNase I was processed for cDNA synthesis using oligo(dT)_20_ primers and SuperScript II reverse transcriptase (Invitrogen). cDNA was then amplified by PCR using HotStar Taq DNA polymerase (Qiagen, Valencia, CA, USA) on a PC815 thermal cycler (Astec, Fukuoka, Japan) at 95°C for 1 minute for denaturation, at 62°C for 40 seconds for annealing, and 72°C for 50 seconds for extension for indicated cycles. PCR cocktails were prepared individually by mixing with the following sense and antisense primer sets: 5′-aagcctactgccttctgttgggac-3′ and 5′-caggcctggctttggctatgacat-3′ for a 153 base pair product of the human *PLD1* gene [NCBI:NM_002662]; 5′-actgagggcagtgccctttgagat-3′ and 5′-tggcaggagctgtgcttctccttt-3′ for a 209 base pair product of the human *PLD2* gene [NCBI:NM_002663]; 5′-tatcccccagccttgagggaagat-3′ and 5′-gagatgcaagtcgccgtattccca-3′ for a 223 base pair product of the human *PLD3* gene [NCBI:NM_012268]; 5′-ggacgtgaaagtcttcatcgtgcc-3′ and 5′-cgtgctgctgaagtaatcctccga-3′ for a 142 base pair product of the human *PLD4* gene [NCBI:NM_138790]; 5′-aagcagagtgcctcaaacctggtc-3′ and 5’tggtgcttgtgctggagataggca-3′ for a 239 base pair product of the human *PLD5* gene [NCBI:NM_152666]; 5’atctctgcctgttcgccttctcca-3′ and 5′-cttgtgatgcatgtagcctgggtc-3′ for a 191 base pair product of the human *PLD6* gene [NCBI:NM_178836]; and 5′-ccatgttcgtcatgggtgtgaacca-3′ and 5′-gccagtagaggcagggatgatgttc-3′ for a 251 base pair product of the glyceraldehyde-3-phosphate dehydrogenase (*G3PDH*) gene [NCBI:NM_002046], serving as a positive control.

For qPCR, cDNA prepared from frozen human brain tissues and a reference RNA of the human frontal cortex (AM6810; Ambion, Invitrogen) was amplified by PCR on LightCycler ST300 (Roche Diagnostics, Tokyo, Japan) using SYBR Green I and the primer sets described above. The expression levels of target genes were standardized against the levels of G3PDH detected in the corresponding cDNA samples. All assays were performed in triplicate.

### Multiple sequence alignment analysis

We performed multiple sequence alignment analysis using CLC Sequence Viewer 7 (CLC bio-Qiagen, Aarhus, Denmark). We imported amino acid sequences of human PLD1 [NCBI:NP_002653.1], PLD2 [NCBI:NP_002654.3], PLD3 [NCBI:NP_036400.2], PLD4 [NCBI:NP_620145.2], PLD5 [NCBI:NP_689879.2], and PLD6 [NCBI:NP_849158.2], and PLD3 orthologs derived from *Bos Taurus* [NCBI:NP_001071509.1], *Mus musculus* [NCBI:NP_035246.1], *Rattus norvegicus* [NCBI:NP_001012167.1], *Danio rerio* [NCBI:XP_003200538.1], *Xenopus laevis* [NCBI:NP_001083260.1], *Drosophila melanogaster* [NCBI:NP_610093.1], and *Caenorhabditis elegans* [NCBI:NP_504824.1]*.*

### Genotyping analysis

Two variants of the human *PLD3* gene, such as rs145999145 comprised of p.Val232Met (p.V223M) corresponding to c.694G > A located in exon 7 and rs4819 comprised of p.Ala442Ala (p.A442A) corresponding to c.1326G > A located in exon 11, were studied by direct sequencing of PCR products amplified from brain cDNA by using sense and antisense primer sets comprised of 5′-cgcatggtggacatgcagaag-3′ and 5′-ggtgtctcttggttgtagcgg-3′ for rs145999145 and 5′-tgctctctctggctgccctgcgtg-3′ and 5′-tcagagcaggcggcaggcgttgcc-3′ for rs4819.

### Coexpression database search

The Coexpression database (COXPRESdb) version 5.0 [22] is freeware of bioinformatics that contains the collection of comprehensive information on gene coexpression retrieved from large sets of public microarray data to estimate gene functions of 11 species, including *Homo sapiens*, *M. musculus*, *R. norvegicus*, *D. melanogaster*, and *C. elegans*[23]. For human, COXPRESdb includes coexpression data derived from 79,948 microarrays, whose raw data are extracted from ArrayExpress and normalized by the robust multi-array average method. COXPRESdb helps us to identify coexpression relationships for a given gene with the probability evaluated by the reliability score and to illustrate the interactive network of the genes surrounding a focused gene on Cytoscape web. By searching on COXPRESdb, we identified the set of genes coexpressed with PLD3, which potentially contain candidates for PLD3 interactors.

### Vector construction, transfection, and immunoprecipitation

The full-length open reading frame of the human *PLD3* gene or the human *GRN* gene, selected as a candidate for PLD3 interactors, was amplified by PCR using PfuTurbo DNA polymerase (Agilent Technologies, Palo Alto, CA, USA) and the set of sense and antisense primers. Subsequently, PCR products were cloned in the expression vector named p3XFLAG-CMV7.1 (Sigma), pCMV-Myc (Clontech, Mountain View, CA, USA), pDsRed-Express-C1 (Clontech), or pZsGreen1-N1 (Clontech) to express a fusion protein with an N-terminal Flag tag, Myc tag, DsRed tag, or C-terminal ZsGreen tag, respectively. The vectors were transfected in HEK293 cells, HeLa cells or SK-N-SH cells using Lipofectamine 2000 reagent (Invitrogen) for transient expression. The M232 isoform of PLD3 was prepared from the cloned vector using QuickChange Lightning Site-Directed Mutagenesis Kit (Agilent Technologies). After cotransfection of the vectors, the protein extract was processed for immunoprecipitation with mouse monoclonal anti-Flag M2 affinity gel (Sigma) or rabbit polyclonal anti-Myc-conjugated agarose (Sigma), followed by western blot with a rabbit polyclonal anti-Myc antibody (Sigma) or a mouse monoclonal anti-FLAG M2 antibody (Sigma), respectively.

### Western blot analysis

To prepare total protein extract, brain tissues and cultured cells were homogenized in RIPA buffer (Sigma) supplemented with a cocktail of protease inhibitors (Sigma), followed by centrifugation at 12,000 rpm for 10 minutes at room temperature to harvest the supernatant. The protein was separated on a 12% SDS-PAGE gel. After gel electrophoresis, the protein was transferred onto nitrocellulose membranes, followed by incubation at room temperature overnight with the anti-PLD3 antibody HPA012800 or a rabbit monoclonal anti-PGRN antibody (EPR3781; Abcam, Cambridge, UK). The membrane was then incubated at room temperature for 30 minutes with horseradish peroxidase-conjugated anti-rabbit IgG (Santa Cruz Biotechnology, Santa Cruz, CA, USA). The specific reaction was visualized by exposing membranes to a chemiluminescent substrate (Thermo Scientific). After the antibody was stripped by incubating membranes at 50°C for 30 minutes in the stripping buffer, composed of 62.5 mM Tris–HCl, pH 6.7, 2% SDS and 100 mM 2-mercaptoethanol, it was processed for relabeling with a goat polyclonal anti-HSP60 antibody (sc-1052; Santa Cruz Biotechnology) or a rabbit polyclonal anti-G3PDH antibody (GTX100118; GeneTex, Irvine, CA, USA), serving as an internal control of protein loading. The signal intensity of the major band was quantified using ImageJ (National Institute of Health, Bethesda, MD, USA). The expression levels of PLD3 were standardized by the corresponding signal intensity of HSP60 or G3PDH.

### Statistical analysis

The statistical significant difference compared between AD (*n* =7) and non-AD (*n* =14) groups was evaluated by Student’s *t* test. *P* <0.05 by the two-tailed test was considered significant.

## Results

### Amino acid sequence homology of human PLD proteins

First, to characterize phylogenetic variation of PLD proteins, we performed multiple sequence alignment analysis. The amino acid sequence of the human PLD3 protein is 96%, 92%, 92%, 58%, 54%, 48%, and 41% identical to the sequences of PLD3 orthologs derived from *B. Taurus*, *M. musculus*, *R. norvegicus*, *D. rerio*, *X. laevis*, *D. melanogaster*, and *C. elegans*, respectively (Additional file 2). All of them contain two copies of the conserved HXKXXXXD/E (HKD) motif, indicating the high level of conservation of PLD3 through evolution. In contrast, the amino acid sequence of the human PLD3 protein showed only 5.7%, 8.5%, 42.5%, 33.9%, and 10.0% identity to the sequences of the human PLD1, PLD2, PLD4, PLD5, and PLD6 proteins, all of which represent PLD3 paralogs (Additional file 3). Thus, we identified PLD4 as the paralog most closely related to PLD3, although the amino acid sequence homology is fairly limited between both.

### Constitutive expression of PLD3 mRNA in human neural cells

**Figure 1:**
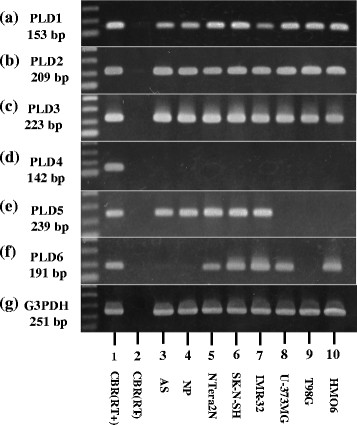
**Constitutive expression of PLD3 mRNA in human neural cells.** mRNA expression was studied by reverse transcription (RT)-polymerase chain reaction in human tissues and cultured cells. **(a)** PLD1, **(b)** PLD2, **(c)** PLD3, **(d)** PLD4, **(e)** PLD5, **(f)** PLD6, and **(g)** glyceraldehyde-3-phosphate dehydrogenase (G3PDH). Lane 1, frontal cortex of the human cerebrum (CBR) with inclusion of the RT step; lane 2, CBR without inclusion of the RT step; lane 3, astrocytes (AS); lane 4, neuronal progenitor (NP) cells; lane 5, NTera2 teratocarcinoma-derived neurons (NTera2N); lane 6, SK-N-SH neuroblastoma; lane 7, IMR-32 neuroblastoma; lane 8, U-373MG glioblastoma; lane 9, T98G glioblastoma; lane 10, HMO6 immortalized microglia. cDNA was amplified by PCR for 35 cycles, except for G3PDH that was amplified for 28 cycles. PLD, phospholipase D.

Next, we studied mRNA expression of PLD3 paralogs in human neural cell lines and tissues by RT-PCR. The human cerebrum, astrocytes, neuronal progenitor cells, NTera2 teratocarcinoma-derived neurons, SK-N-SH neuroblastoma, IMR-32 neuroblastoma, U-373MG glioblastoma, T98 glioblastoma, and HMO6 immortalized microglia constitutively expressed PLD1, PLD2, and PLD3 transcripts (Figure 1a,b,c, lanes 1, 3 to 10). In contrast, more limited cell and tissue types expressed PLD4, PLD5, and PLD6 transcripts (Figure 1d,e,f, lanes 1, 3 to 10). G3PDH, a housekeeping gene serving as a positive control, was detected in all cells and tissues examined (Figure 1g, lanes 1, 3 to 10), while no products were amplified when the reverse transcription step was omitted (Figure 1a to g, lane 2). We thus found that PLD1, PLD2 and PLD3 are expressed constitutively in a wide range of human neural cells.

### Reduced expression of PLD3 mRNA and protein in Alzheimer’s disease brains

Next, we studied PLD3 mRNA and protein expression in frozen tissues of the frontal cortex, derived from four patients that died from non-neurological causes, six amyotrophic lateral sclerosis patients, four Parkinson’s disease patients, and seven AD cases (Table 1). Before starting this, we determined the genotype of rs145999145 and rs4819 of the human *PLD3* gene in all samples. The nonsynonymous single nucleotide polymorphism rs145999145 comprised of p.V232M corresponding to c.694G > A in exon 7 represents the most significant AD risk-associated variant among several single nucleotide polymorphisms on *PLD3*, on which the M232 isoform confers a doubled risk for development of late-onset AD [5]. However, in our series, all AD and non-AD cases exhibited the V232 isoform (Table 1; Additional file 4). The synonymous single nucleotide polymorphism rs4819 comprised of p.A442A corresponding to c.1326G > A in exon 11 affects alternative splicing, leading to reduced expression of exon 11 containing transcripts [5]. In our series, all AD and non-AD cases exhibited the common allele c.1326G (Table 1). These results suggested that AD risk-associated variants on rs145999145 and rs4819 are extremely rare even in AD patients, consistent with the genotyping data of the cohorts participating in the NHLBI Exome Sequencing Project.

**Figure 2:**
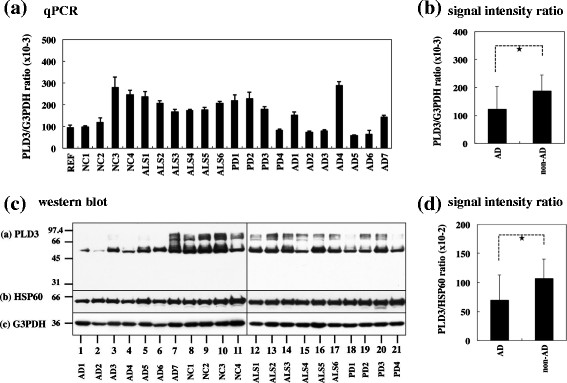
**Reduced expression of PLD3 mRNA and protein in Alzheimer’s disease brains. (a)**, **(b)** PLD3 mRNA expression. mRNA expression was studied by quantitative reverse transcription-polymerase chain reaction (qPCR) in human brain tissues derived from a reference of the human frontal cortex (REF), four non-neurological controls (NC), six amyotrophic lateral sclerosis (ALS) patients, four Parkinson’s disease (PD) patients, and seven Alzheimer’s disease (AD) cases. PLD3 expression levels were standardized against those of glyceraldehyde-3-phosphate dehydrogenase (G3PDH), and the statistical significance in difference between AD and non-AD groups was evaluated by Student’s *t* test. *P =0.0499 (PLD3 vs. G3PDH). **(c)**, **(d)** PLD3 protein expression. Protein expression was studied by western blot in human brain tissues derived from four NC, six ALS patients, four PD patients, and seven AD cases. PLD3 expression levels were standardized against those of HSP60 or G3PDH, and the statistical significance in difference between AD and non-AD groups was evaluated by Student’s *t* test. *P =0.0416 (PLD3 vs. HSP60). PLD, phospholipase D.

By qPCR, AD cases showed reduced PLD3 mRNA levels, when compared with the levels in non-AD cases at a marginal significance level (*P* =0.0499) (Figure 2a,b). By western blot using HPA012800, whose specificity was validated by immunoblot of recombinant PLD3 expressed in HEK293 cells (Additional file 1), AD cases showed significantly reduced PLD3 protein levels, when compared with the levels in non-AD cases following standardization against HSP60 (*P* =0.0416) (Figure 2c,d). However, this difference did not reach statistical significance when PLD3 protein levels were standardized against those of G3PDH (*P* =0.1644). Taken together, we tentatively concluded that PLD3 expression is reduced in AD brains but modestly at both mRNA and protein levels.

### Accumulation of PLD3 immunoreactivity in neuritic plaques in Alzheimer’s disease brains

**Figure 3:**
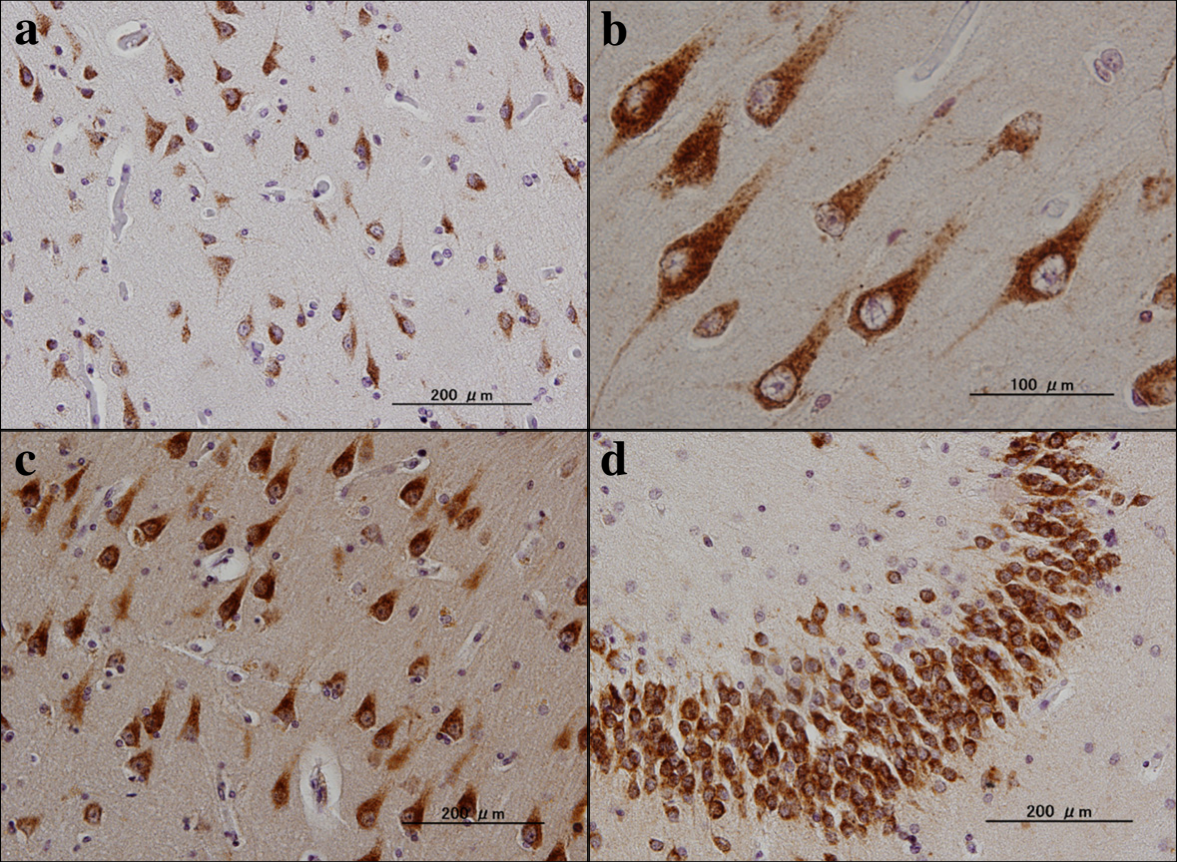
**PLD3 immunoreactivity in non-Alzheimer’s disease brains.** PLD3 immunoreactivity was studied in non-Alzheimer’s disease brains by immunohistochemistry. **(a)** Non-neurological control, the frontal cortex, neuronal cytoplasmic labeling. **(b)** Non-neurological control, the hippocampal CA1 region, neuronal cytoplasmic labeling. **(c)** Parkinson’s disease patient, the hippocampal CA1 region, neuronal cytoplasmic labeling. **(d)** Amyotrophic lateral sclerosis patient, the hippocampal dentate gyrus, neuronal cytoplasmic labeling. PLD, phospholipase D.

**Figure 4:**
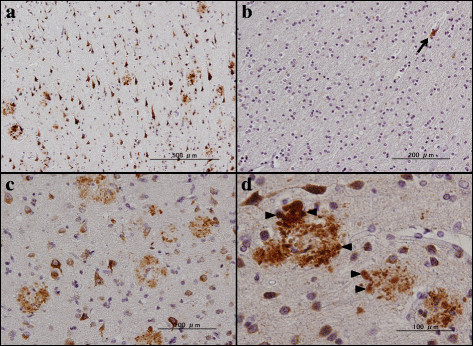
**PLD3 immunoreactivity in Alzheimer’s disease brains (I).** PLD3 immunoreactivity was studied in Alzheimer’s disease brains by immunohistochemistry. **(a)** to **(d)** Numerous senile plaques containing dystrophic neurites expressing intense PLD3 immunoreactivity in the frontal cortex. Arrowheads in (d) represent swollen neurites in senile plaques. Arrow in (b) indicates pericyte cytoplasmic labeling in the white matter. PLD, phospholipase D.

Next, by immunohistochemistry using HPA012800, we studied the expression of PLD3 immunoreactivity in the frontal cortex and the hippocampus of 10 AD cases and 13 non-AD cases, composed of four non-neurological cause controls, four amyotrophic lateral sclerosis patients, three Parkinson’s disease patients, and two multiple system atrophy cases (Table 1). In all cases, nearly all cortical neurons, hippocampal pyramidal neurons and dentate gyrus granule cells expressed intense PLD3 immunoreactivity with the location in the cytoplasm by forming fine granular structures, enriched in the soma and in proximal neurites (Figure 3a,b,c,d). A subset of pericytes surrounding capillaries also expressed intense PLD3 immunoreactivity (Figure 4b). Notably, numerous senile plaques containing swollen dystrophic neurites expressed intense PLD3 immunoreactivity in the frontal cortex of AD brains (Figure 4a,c,d). In contrast, PLD3-labeled senile plaques were barely found in non-AD brains (data not shown). PLD3 immunoreactivity on neurons and senile plaques was completely abolished, when the tissue sections were incubated with the HPA012800 antibody preabsorbed by a recombinant PLD3 fragment spanning amino acid residues 93 to 218 (Additional file 5).

**Figure 5:**
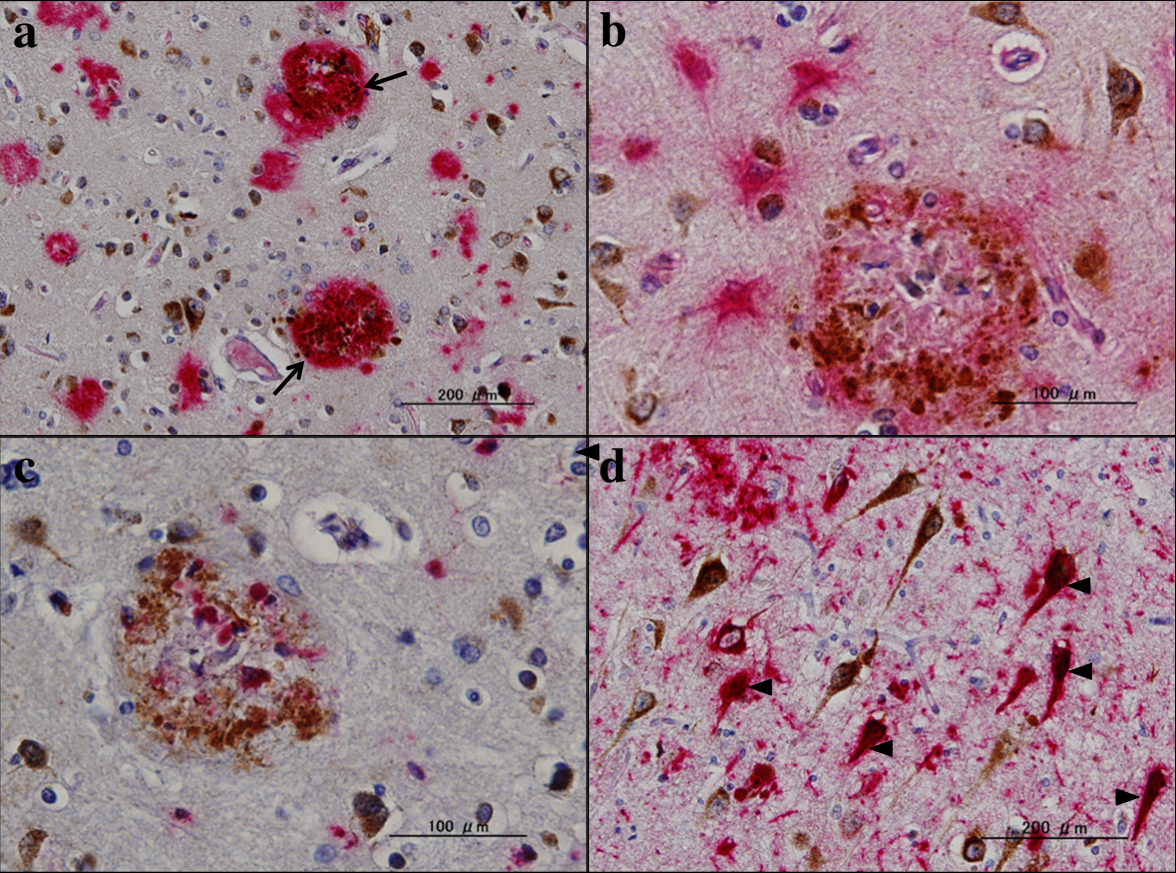
**PLD3 immunoreactivity in Alzheimer’s disease brains (II).** PLD3 immunoreactivity was studied in Alzheimer’s disease brains by double-labeling immunohistochemistry. **(a)** Frontal cortex, PLD3 (brown), amyloid beta (Aβ; red), colocalization of PLD3 and Aβ (arrows). **(b)** Frontal cortex, PLD3 (brown), GFAP (red), reactive astrocytes do not express PLD3. **(c)** Frontal cortex, PLD3 (brown), CD68 (red), activated microglia do not express PLD3. **(d)** Hippocampal CA1 region, PLD3 (brown), AT8-tau (red), colocalization of neurofibrillary tangles and PLD3 (arrowheads). PLD, phospholipase D.

**Figure 6:**
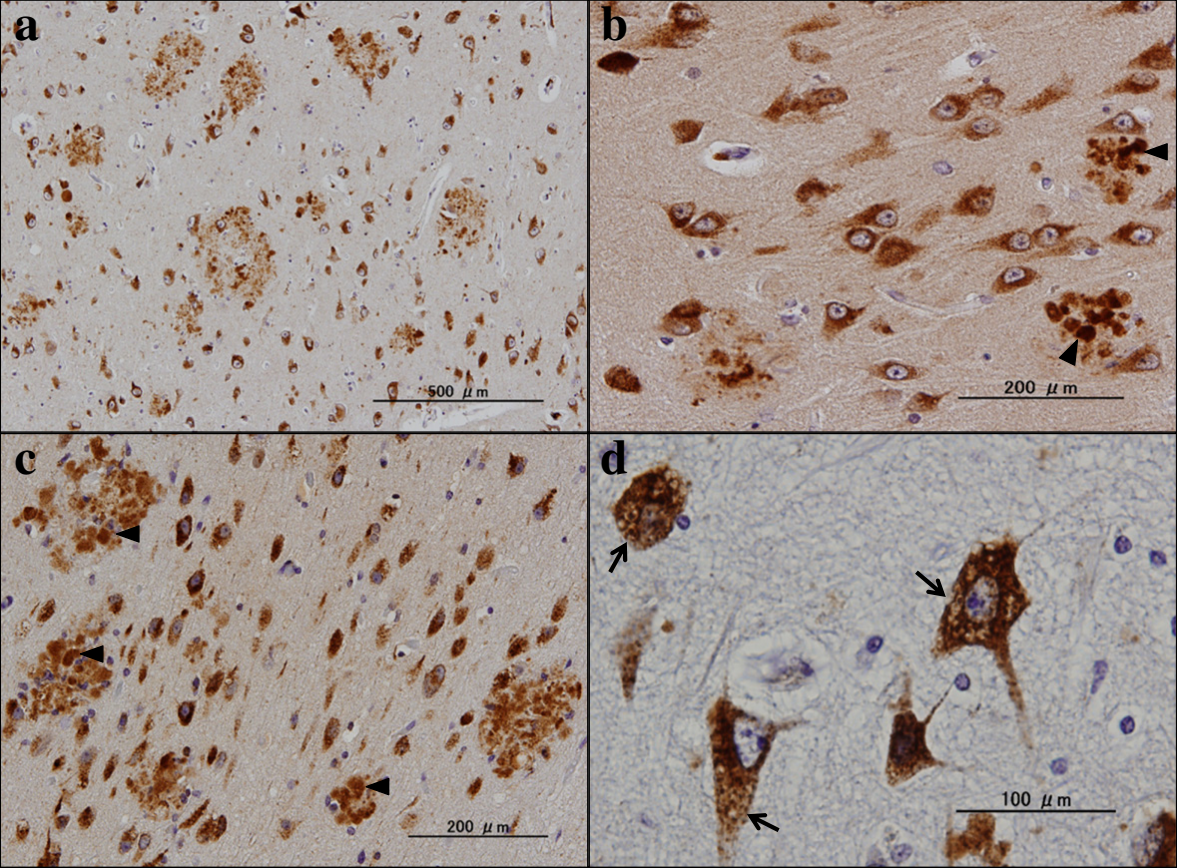
**PLD3 immunoreactivity in Alzheimer’s disease brains (III).** PLD3 immunoreactivity was studied in Alzheimer’s disease brains by immunohistochemistry. **(a)** to **(d)** Senile plaques containing dystrophic neurites expressing intense PLD3 immunoreactivity in the hippocampal CA1 region. Arrowheads in **(b)** and **(c)** represent swollen neurites in senile plaques. Arrows in **(d)** indicate granulovaculoar degeneration in hippocampal CA1 neurons. PLD, phospholipase D.

By double labeling, senile plaques reactive for PLD3 were decorated with Aβ immunoreactivity (Figure 5a). In contrast, oligodendocytes located in the white matter did not express PLD3 (Figure 4b). Furthermore, reactive astrocytes and activated microglia accumulated in the center of senile plaques and surrounding them did not express PLD3 (Figure 5b,c). Senile plaques reactive for PLD3 were often accompanied by loss of PLD3-positive cortical neurons (Figure 4c). In the hippocampus of AD brains, senile plaques containing swollen dystrophic neurites expressed intense PLD3 immunoreactivity, often accompanied by loss of PLD3-positive pyramidal neurons (Figure 6a,b,c). AT8-positive neurofibrillary tangles in the hippocampal neurons were frequently decorated with PLD3 immunoreactivity (Figure 5d), whereas the vacuoles of granulovacuolar degeneration, which are most often found in the hippocampal CA1 region of AD brains, were devoid of PLD3 immunoreactivity (Figure 6d). We thus found that PLD3 immunoreactivity is accumulated on neuritic plaques in AD brains.

### Molecular interaction of PLD3 with progranulin

**Figure 7:**
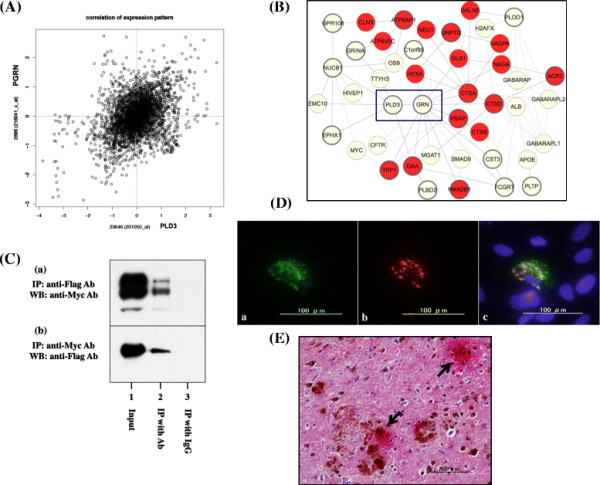
**Coexpression and molecular interaction of PLD3 with progranulin. (A), (B)** Coexpression of PLD3 and progranulin (PGRN). The genes coexpressed with PLD3 were identified by database search on COXPRESdb [23] and are listed in Additional file 6. (A) Correlation coefficient of gene expression between PLD3 (*x* axis indicates probe ID 201050_at) and PRGN (*y* axis indicates probe ID 216041_x_at) is 0.454. (B) Interactive network of the set of genes coexpressed with PLD3 shows an enrichment of lysosomal proteins (nodes indicated in red). The interaction between PLD3 and GRN is highlighted by a blue square. **(C)** Coimmunoprecipitation of PLD3 and PGRN. Myc-tagged PLD3 and Flag-tagged PGRN were coexpressed in HEK293 cells. The protein extract was processed for **(a)** immunoprecipitation (IP) with anti-Flag antibody followed by western blot (WB) with anti-Myc antibody or **(b)** IP with anti-Myc antibody followed by WB with anti-Flag antibody. Lane 1, input control; lane 2, IP with the specific antibody; lane 3, IP with normal IgG. **(D)** Colocalization of PLD3 and PGRN in cultured cells. ZsGreen-tagged PLD3 and DsRed-tagged PGRN were coexpressed in SK-N-SH cells. **(a)** PLD3, **(b)** PGRN, and **(c)** merge. **(E)** Colocalization of PLD3 and PGRN in Alzheimer’s disease (AD) brains. The coexpression of PLD3 (brown) and PGRN (red) was studied in the frontal cortex of AD brains by double-labeling immunohistochemistry. Arrows indicate the prominent accumulation of PLD3 and PGRN on neuritic plaques. Ab, antibody; PLD, phospholipase D.

Next, to investigate the role of PLD3 in AD pathology, we attempted to identify a potential interactor for PLD3 by the bioinformatics approach. By searching the genes coexpressed with PLD3 on COXPRESdb [23], we identified the human *GRN* gene encoding PGRN, whose mutation is responsible for frontotemporal lobar degeneration (FTLD) and neuronal ceroid lipofuscinosis [24], ranked as the second most significant gene coexpressed with PLD3 (Additional file 6). The correlation coefficient of gene expression between PLD3 and PGRN exceeds 0.4, suggesting a definite positive correlation between both (Figure 7A). Furthermore, previous studies indicated that several *GRN* variants serve as a risk factor for AD [25],[26]. Thereafter, we focused our attention on *GRN* (PGRN) as the most promising candidate for PLD3 interactors in the present study. The database search also showed that the set of genes coexpressed with PLD3 constitute the molecular network enriched with lysosomal proteins (Figure 7B). By coimmunoprecipitation experiments, we validated a direct interaction between Myc-tagged PLD3 and Flag-tagged PGRN (Figure 7C,a,b). Furthermore, we identified a partial overlap of ZsGreen-tagged PLD3 and DsRed-tagged PGRN in cytoplasmic granular structures of SK-N-SH cells (Figure 7D,a to c). By double-labeling immunohistochemistry, we identified coexpression of PLD3 and PGRN on neuritic plaques in AD brains (Figure 7E). Taken together, we found that PLD3 is coexpressed and interacts with PGRN both in cultured cells *in vitro* and in AD brains *in vivo*.

Finally, we studied biological effects of PLD3 on PGRN protein expression in SK-N-SH cells. Transient overexpression of Flag-tagged PLD3, either V232 or M232, or GFP as a negative control did not alter PGRN protein levels (Figure S6a,b,c,d, lanes 1 to 4 in Additional file 7). These results suggested that PLD3 is unlikely to act as a direct regulator of PGRN protein expression.

## Discussion

A recent whole-exome sequencing study showed that a rare variant rs145999145 composed of p.V232M located in exon 7 of the *PLD3* gene confers a doubled risk for late-onset AD [5]. However, the precise cellular location and distribution of PLD3 in AD brains remain largely unknown. We studied PLD3 expression patterns and levels in a series of AD and control brains by qPCR, western blot, immunohistochemistry, and bioinformatics analysis. We found that the levels of PLD3 mRNA and protein expression are reduced in AD brains but modestly, consistent with the previous observations showing that PLD3 mRNA levels are decreased in AD brains based on the public microarray dataset numbered GSE5281 [5]. PLD3 is expressed constitutively in human neural cell lines, and cortical neurons, hippocampal pyramidal and granular neurons, and vascular pericytes but not in glial cells in human brains. We for the first time showed that PLD3 immunoreactivity is accumulated in dystrophic neurites on senile plaques, most prominently in AD brains, regardless of reduced levels of PLD3. We assume that reduced expression of PLD3 is attributable to a profound decrease in total populations of neurofilament-positive neurons in AD brains, as reported recently [19].

By bioinformatics approach, we identified PRGN as one of the most significant genes coexpressed with PLD3 on COXPRESdb, a comprehensive database of gene coexpression [23]. We found that PLD3 is actually coexpressed and interacts with PGRN both in cultured cells *in vitro* and in AD brains *in vivo*. A previous study showed that PGRN acts as a chemotactic factor for microglia and PGRN-treated microglia exhibit an enhanced capacity to phagocytose Aβ1–42 [27]. Recently, we found that senile plaques, neurofibrillary tangles, and the perivascular neuropil express PGRN immunoreactivity in AD brains [19]. Supporting this, PGRN expression is enhanced on dense core amyloid plaques in a transgenic mouse model of AD [28]. Interestingly, PLD3 mRNA expression is decreased in the brains of the patients with *GRN*-mutated FTLD with ubiquitinated TAR DNA-binding protein-43-positive inclusions [29]. These observations suggest that PLD3, coexpressed with PRGN, plays a key role in senile plaque formation and neurodegeneration in AD brains.

At present, the precise biological function of PLD3 remains unknown in the human central nervous system. We found that PLD3 orthologs are highly conserved through evolution from the human to the worm, and that PLD4 is the paralog most closely related to PLD3. However, PLD3 but not PLD4 is expressed constitutively in various human neural cell lines, suggesting a housekeeping function of PLD3 required for the maintenance of vital cellular processes. For example, PLD3 is identified as one of irradiation-responsive genes in human lymphoblastoid cells, serving as a genetic modifier for breast cancer susceptibility genes BRCA1 and BRCA2 [30], suggesting a role of PLD3 in DNA damage response.

We found that the set of genes coexpressed with PLD3 constitute a lysosomal protein network. In contrast, previous studies showed that PLD3 is a type 2 transmembrane glycoprotein primarily located in ER [18],[31]. PLD3 expression levels are elevated in mouse C2C12 cells following ER stress [31]. PGRN is accumulated in the ER and the Golgi complex in mouse cortical neurons and microglia [32]. These observations suggest that PLD3 interacts with PRGN in the ER compartment in human neurons. Interestingly, PLD3 is colocalized with lysosomal-associated membrane protein 2 (LAMP2), an endosome/lysosome marker in HeLa cells [33]. Sortilin, serving as a cell-surface receptor for PGRN, regulates trafficking and targeting of PGRN to lysosomes [34]. Therefore, an alternative possibility exists that PLD3 cooperates physiologically with PRGN in the lysosomal compartment in human neurons. Starvation stress transports PLD1 located principally in late endosomes and the Golgi complex to the outer membrane of microtubule-associated protein 1 light chain 3-positive amphisome, an autophagic vacuole formed by fusion of an autophagosome and an endosome, suggesting a pivotal role for PLD1 in the regulation of autophagy [35]. These observations suggest that subcellular location of PLD enzymes is changeable, depending on cell types and cellular microenvironments in the context of interacting proteins. We could therefore propose a working hypothesis that PLD3 transported from ER to endosome/lysosome/autophagosome compartments might play a pivotal role in regulation of autophagy.

The database search of rs145999145 composed of p.V232M located in exon 7 of the *PLD3* gene on the Polymorphism Phenotyping v2 (Polyphen-2) program [36] indicates that this variant produces a potentially deleterious effect on protein function [5]. By PCR-based direct sequencing, we could not find the AD risk-associated variant M232 in any AD and non-AD cases examined. However, even a subtle decrease in PLD3 expression and function could cause production of greater amounts of amyloidogenic species of Aβ, in view of the evidence that PLD3 acts as a negative regulator of APP processing [5]. Since PLD3 is coexpressed with PGRN, the possibility exists that PLD3 acts as a positive regulator of PRGN production – as transmembrane protein 106B (TMEM106B), one of the risk genes for FTLD with ubiquitinated TAR DNA-binding protein-43-positive inclusions, does [37]. With the respect to the *GRN* gene, a partial loss of function causes FTLD with ubiquitinated TAR DNA-binding protein-43-positive inclusions, while the complete loss of function develops neuronal ceroid lipofuscinosis [24]. We therefore studied direct effects of PLD3 on PGRN protein expression in SK-N-SH cells. However, we found that overexpression of PLD3, either the V232 or M232 isoform, does not affect PGRN protein expression, suggesting that PLD3 does not act as a direct regulator of PGRN protein production.

## Conclusions

The levels of PLD3 mRNA and protein expression were reduced in AD brains, consistent with previous observations [5]. Notably, we for the first time identified an intense accumulation of PLD3 on neuritic plaques coexpressed with PGRN in AD brains, suggesting the possibility that PLD3 plays a key role in the pathological processes of AD.

## Abbreviations

Aβ: amyloid beta

AD: Alzheimer’s disease

APP: amyloid-beta precursor protein

COXPRESdb: Coexpression database

ER: endoplasmic reticulum

FTLD: frontotemporal lobar degeneration

G3PDH: glyceraldehyde-3-phosphate dehydrogenase

PGRN: progranulin

PLD3: phospholipase D3

qPCR: quantitative reverse transcription-polymerase chain reaction

## Competing interests

The authors declare that they have no competing interests.

## Authors’ contributions

JS and KA designed the study. JS, YK, NK, and YY carried out qPCR, western blot, immunohistochemistry, and genetic analysis. TI, YS, and KA validated the pathological diagnosis of autopsied brains. JS drafted the manuscript. YK, NK, YY, TI, YS, and KA have read the draft and approved the final manuscript.

## Additional files

## Supplementary Material

Additional file 1 **Figure S1 showing characterization of anti-PLD3 antibody.** The full-length open reading frame of the human PLD3 gene cloned in the vector expressing a fusion protein with an N-terminal Flag tag was transiently expressed in HeLa cells. The protein extract was processed for western blot. **(a)** PLD3, the HPA012800 antibody, **(b)** Flag, and **(c)** HSP60, an internal control for protein loading. Lanes 1 and 2 represent the protein extract of (1) non-transfected cells and (2) cells expressing PLD3. The Flag-tagged PLD3 protein is expressed as three distinct bands possibly derived from differential glycosylation [18]. Click here for file

Additional file 2 **Figure S2 showing multiple sequence alignment of PLD3 orthologs.** Multiple sequence alignment analysis was performed by importing the corresponding amino acid sequences into CLC Sequence Viewer 7. The sequences are derived from the PLD3 protein of *Homo sapiens*, *Bos Taurus*, *Mus musculus*, *Rattus norvegicus*, *Danio rerio*, *Xenopus laevis, Drosophila melanogaster*, and *Caenorhabditis elegans*. The conserved HXKXXXXD/E (HKD) motifs are highlighted by the red line. Click here for file

Additional file 3 **Figure S3 showing multiple sequence alignment of PLD3 paralogs.** Multiple sequence alignment analysis was performed by importing the corresponding amino acid sequences into CLC Sequence Viewer 7. The sequences are derived from the human PLD1, PLD2, PLD3, PLD4, PLD5, and PLD6 proteins. The conserved HXKXXXXD/E (HKD) motifs are highlighted by the red line. Click here for file

Additional file 4 **Figure S4 showing genotyping analysis of p.Val232Met.** The genotype of rs145999145 (c.694G>A, p.V232M) located in exon 7 of the human *PLD3* gene was studied by direct sequencing of PCR products amplified from brain cDNA. **(a)** to **(d)** indicate V232/V232 homozygote (underline) of representative cases of (a) non-neurological controls (NC), (b) amyotrophic lateral sclerosis (ALS) patients, (c) Parkinson’s disease (PD) patients, and (d) AD cases. Click here for file

Additional file 5 **Figure S5 showing validation of the specificity of anti-PLD3 antibody by immunoabsorption.** PLD3 immunoreactivity was studied in the frontal cortex of AD brains by immunohistochemistry. **(a)**, **(b)** Immunolabeling with (a) the HPA012800 antibody and (b) the antibody preabsorbed by a recombinant PLD3 fragment spanning amino acid residues 93 to 218. Click here for file

Additional file 6 Table S1 presenting the top 20 significant genes coexpressed with PLD3 on COXPRESdb. Click here for file

Additional file 7 **Figure S6 showing PLD3 overexpression did not alter the levels of PRGN protein expression in SK-N-SH cells.** Flag-tagged GFP or PLD3, either V232 or M232, was transiently expressed for 48 hours in SK-N-SH cells. The protein extract was then processed for western blot. **(a)** PLD3, **(b)** Flag, **(c)** PGRN, and **(d)** Hsp60, as an internal control for protein loading. Lanes 1 to 4 represent the protein extract of (1) nontransfected cells, and the cells expressing (2) GFP as a negative control, (3) PLD3-V232, and (4) PLD3-M232. Click here for file
