# Supplementary figures and images for: PLD3 is accumulated on neuritic plaques in Alzheimer’s disease brains
Source: Alzheimers Res Ther. 2014 Nov 2;6(9):70. doi: 10.1186/s13195-014-0070-5 (PMC4255636; doi:10.1186/s13195-014-0070-5)

## Slide 1
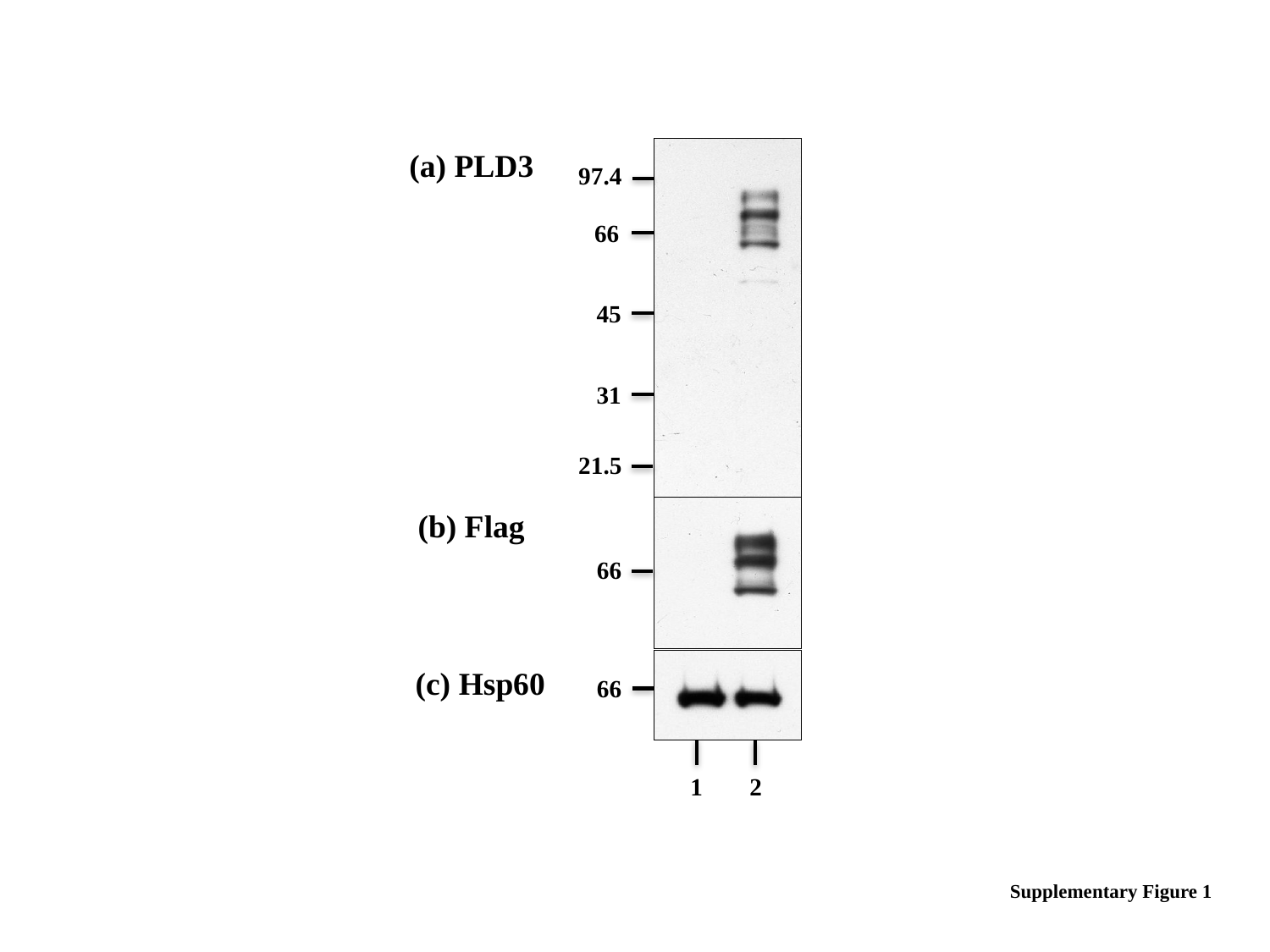

(a) PLD3
97.4
66
45
31
21.5
(b) Flag
66
(c) Hsp60
66
1
2
Supplementary Figure 1

Supplement: Additional file 1 — Figure S1 showing characterization of anti-PLD3 antibody. The full-length open reading frame of the human PLD3 gene cloned in the vector expressing a fusion protein with an N-terminal Flag tag was transiently expressed in HeLa cells. The protein extract was processed for western blot. (a) PLD3, the HPA012800 antibody, (b) Flag, and (c) HSP60, an internal control for protein loading. Lanes 1 and 2 represent the protein extract of (1) non-transfected cells and (2) cells expressing PLD3. The Flag-tagged PLD3 protein is expressed as three distinct bands possibly derived from differential glycosylation [18]. [file s13195-014-0070-5-S1.pptx]

## Slide 1
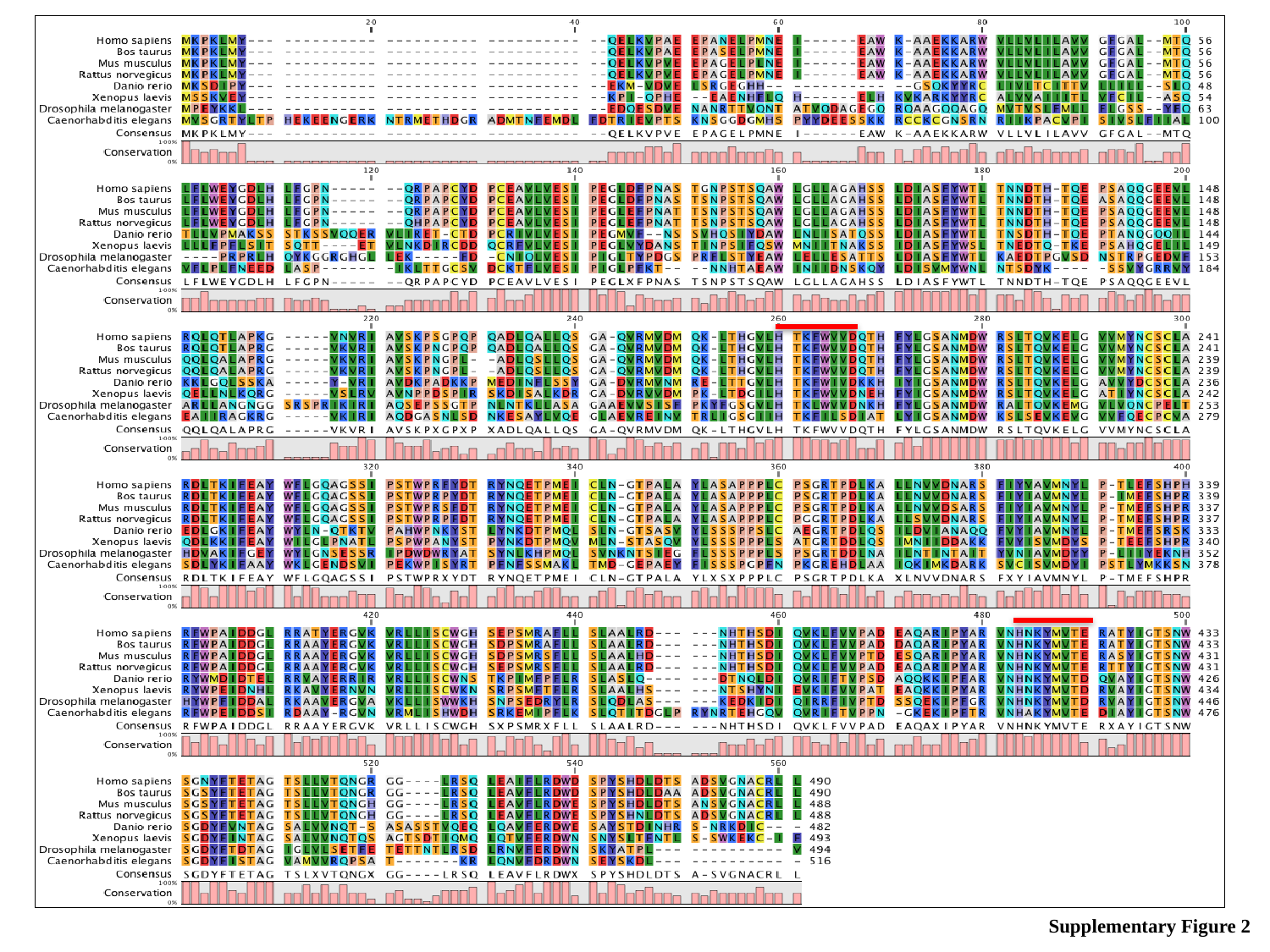

Supplementary Figure 2

Supplement: Additional file 2 — Figure S2 showing multiple sequence alignment of PLD3 orthologs. Multiple sequence alignment analysis was performed by importing the corresponding amino acid sequences into CLC Sequence Viewer 7. The sequences are derived from the PLD3 protein of Homo sapiens, Bos Taurus, Mus musculus, Rattus norvegicus, Danio rerio, Xenopus laevis, Drosophila melanogaster, and Caenorhabditis elegans. The conserved HXKXXXXD/E (HKD) motifs are highlighted by the red line. [file s13195-014-0070-5-S2.pptx]

## Slide 1
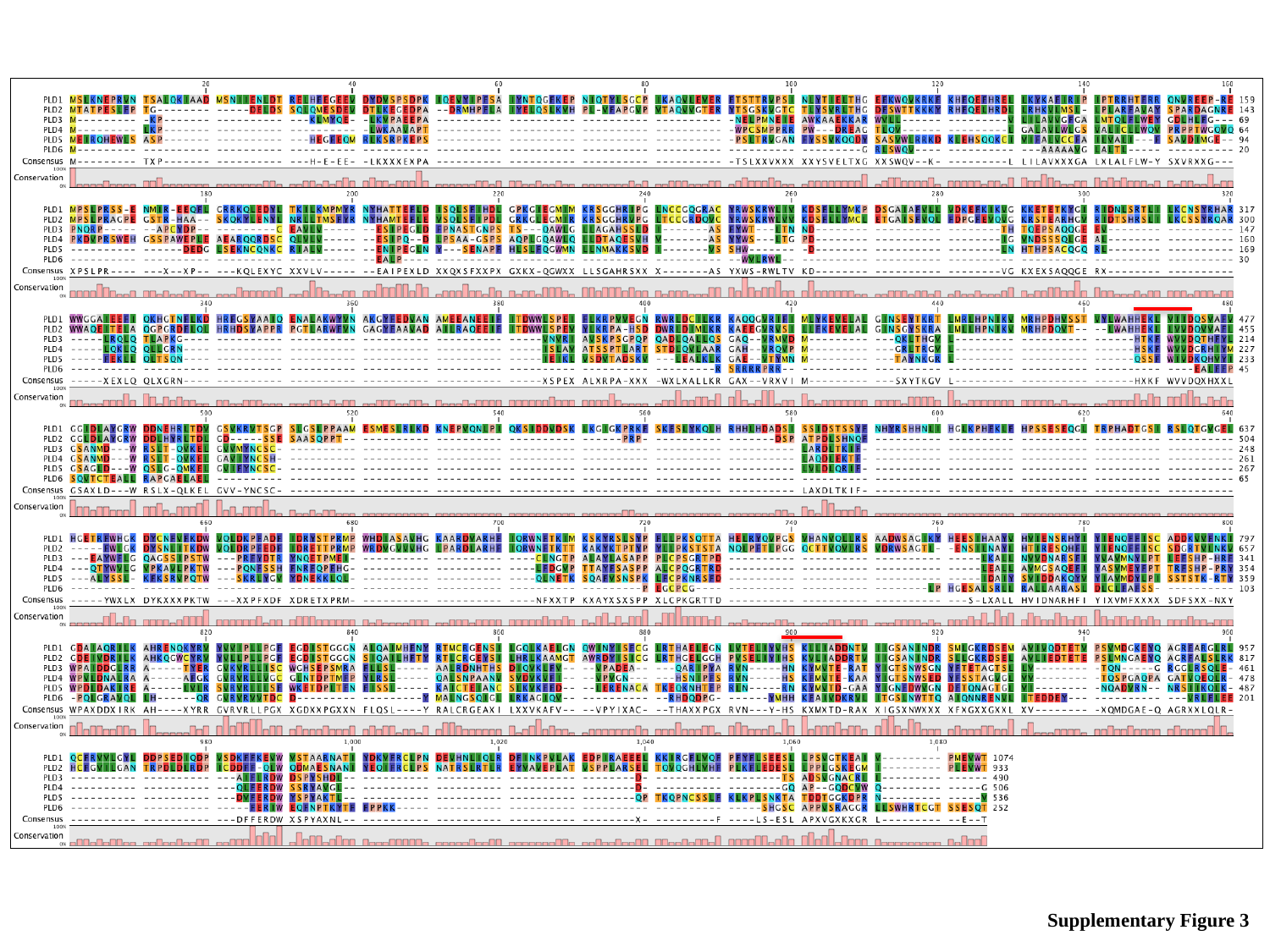

Supplementary Figure 3

Supplement: Additional file 3 — Figure S3 showing multiple sequence alignment of PLD3 paralogs. Multiple sequence alignment analysis was performed by importing the corresponding amino acid sequences into CLC Sequence Viewer 7. The sequences are derived from the human PLD1, PLD2, PLD3, PLD4, PLD5, and PLD6 proteins. The conserved HXKXXXXD/E (HKD) motifs are highlighted by the red line. [file s13195-014-0070-5-S3.pptx]

## Slide 1
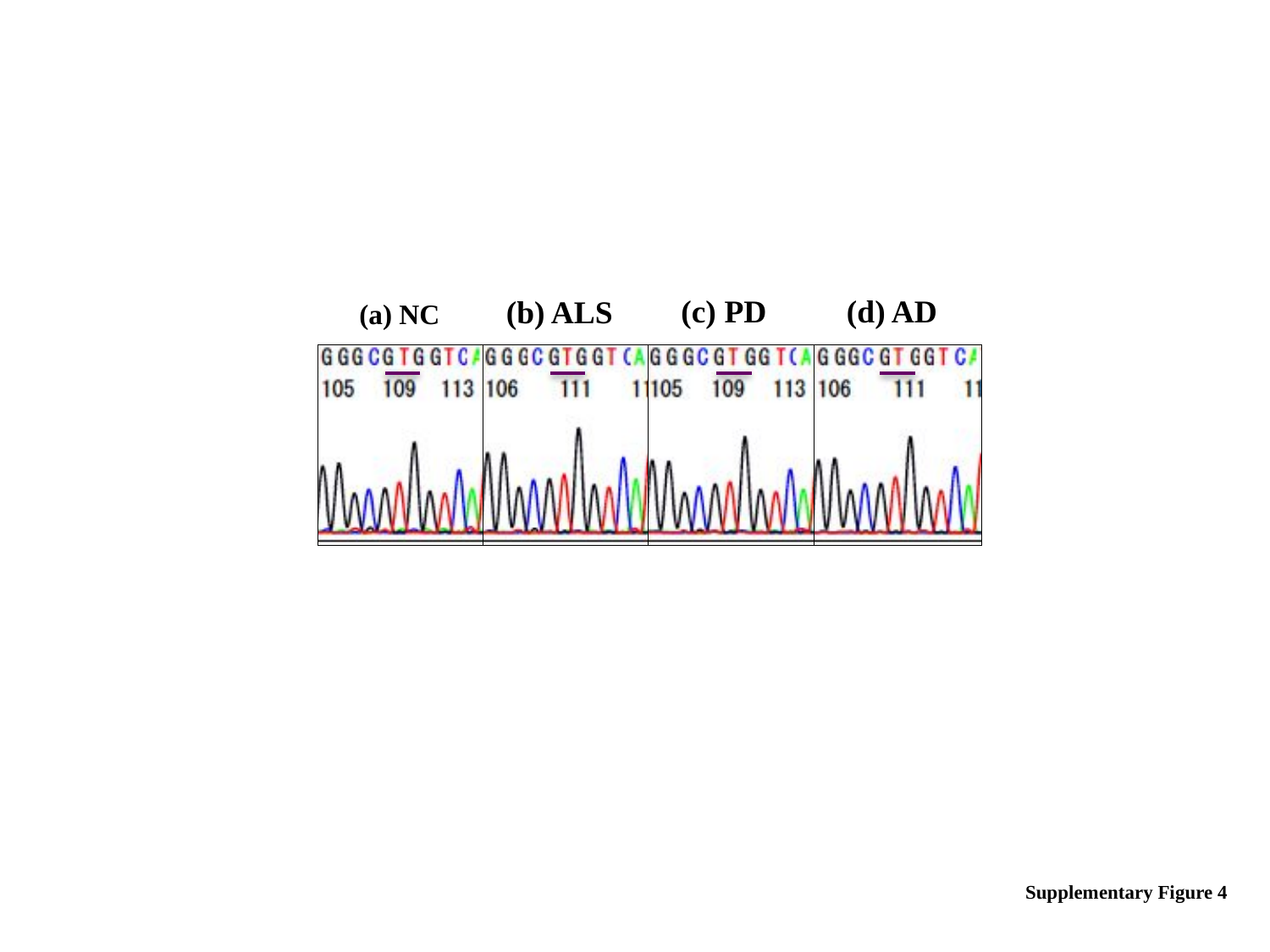

(c) PD
(d) AD
(b) ALS
(a) NC
Supplementary Figure 4

Supplement: Additional file 4 — Figure S4 showing genotyping analysis of p.Val232Met. The genotype of rs145999145 (c.694G>A, p.V232M) located in exon 7 of the human PLD3 gene was studied by direct sequencing of PCR products amplified from brain cDNA. (a) to (d) indicate V232/V232 homozygote (underline) of representative cases of (a) non-neurological controls (NC), (b) amyotrophic lateral sclerosis (ALS) patients, (c) Parkinson’s disease (PD) patients, and (d) AD cases. [file s13195-014-0070-5-S4.pptx]

## Slide 1
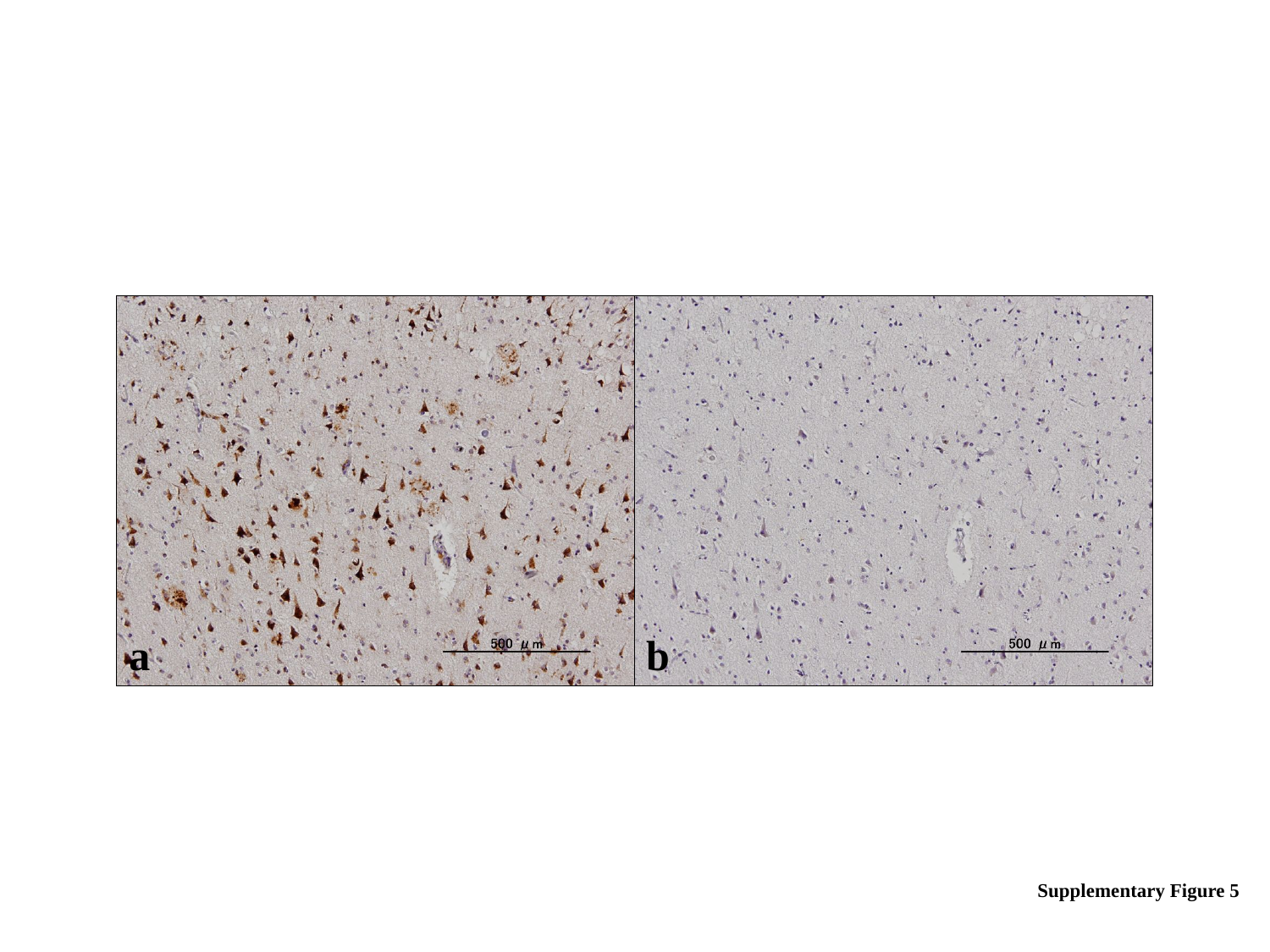

a
b
Supplementary Figure 5

Supplement: Additional file 5 — Figure S5 showing validation of the specificity of anti-PLD3 antibody by immunoabsorption. PLD3 immunoreactivity was studied in the frontal cortex of AD brains by immunohistochemistry. (a), (b) Immunolabeling with (a) the HPA012800 antibody and (b) the antibody preabsorbed by a recombinant PLD3 fragment spanning amino acid residues 93 to 218. [file s13195-014-0070-5-S5.pptx]

## Slide 1
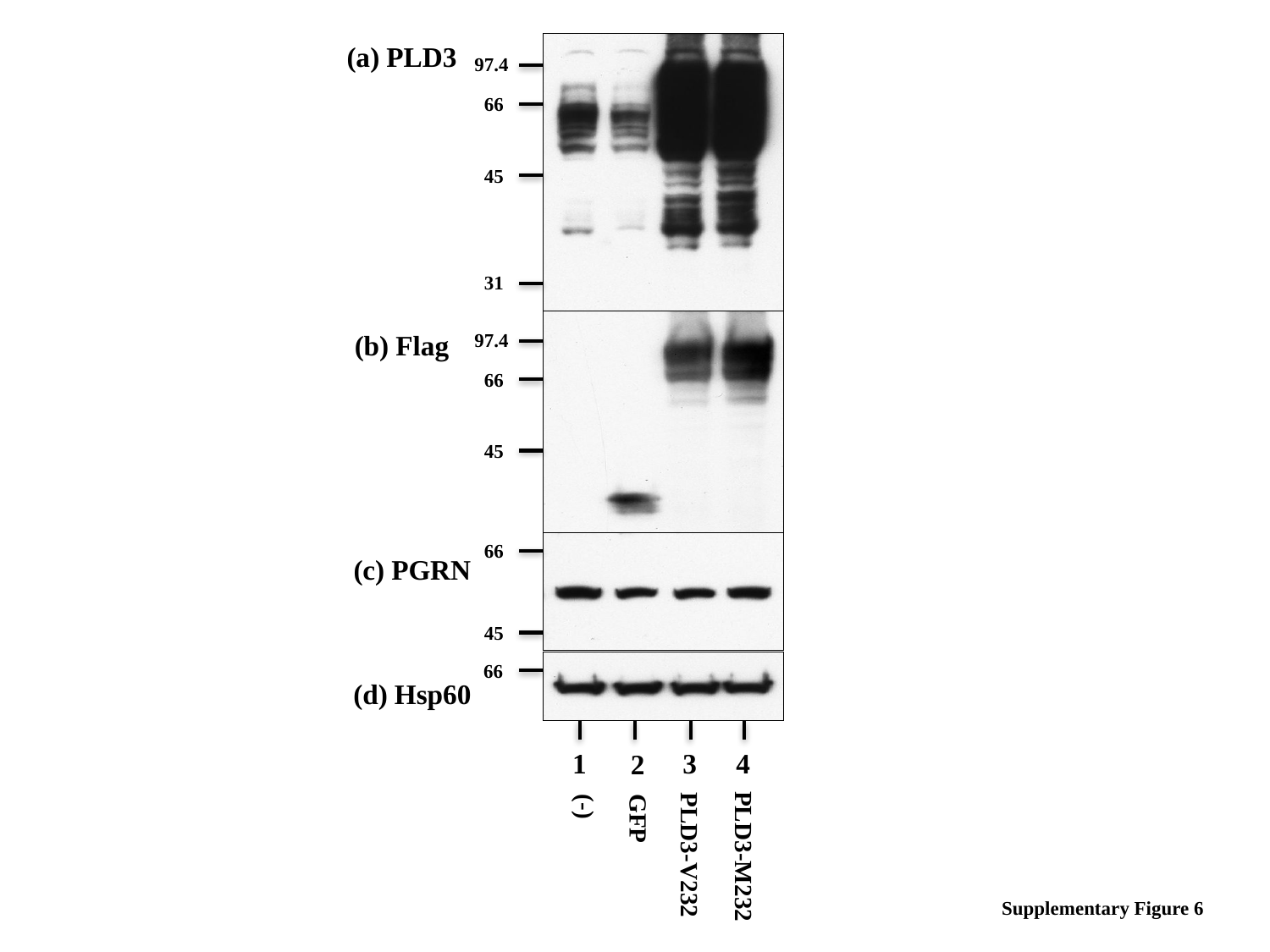

(a) PLD3
97.4
66
45
31
(b) Flag
97.4
66
45
66
(c) PGRN
45
66
(d) Hsp60
3
4
1
2
(-)
GFP
PLD3-V232
PLD3-M232
Supplementary Figure 6

Supplement: Additional file 7 — Figure S6 showing PLD3 overexpression did not alter the levels of PRGN protein expression in SK-N-SH cells. Flag-tagged GFP or PLD3, either V232 or M232, was transiently expressed for 48 hours in SK-N-SH cells. The protein extract was then processed for western blot. (a) PLD3, (b) Flag, (c) PGRN, and (d) Hsp60, as an internal control for protein loading. Lanes 1 to 4 represent the protein extract of (1) nontransfected cells, and the cells expressing (2) GFP as a negative control, (3) PLD3-V232, and (4) PLD3-M232. [file s13195-014-0070-5-S7.pptx]
